# Clinical and Pharmacological Investigation of Myotoxicity in Sri Lankan Russell’s Viper (*Daboia russelii*) Envenoming

**DOI:** 10.1371/journal.pntd.0005172

**Published:** 2016-12-02

**Authors:** Anjana Silva, Christopher Johnston, Sanjaya Kuruppu, Daniela Kneisz, Kalana Maduwage, Oded Kleifeld, A. Ian Smith, Sisira Siribaddana, Nicholas A. Buckley, Wayne C. Hodgson, Geoffrey K. Isbister

**Affiliations:** 1 Monash Venom Group, Department of Pharmacology, Biomedicine Discovery Institute, Monash University, Melbourne, Victoria, Australia; 2 Faculty of Medicine and Allied Sciences, Rajarata University of Sri Lanka, Saliyapura, Sri Lanka; 3 South Asian Clinical Toxicology Research Collaboration, University of Peradeniya, Peradeniya, Sri Lanka; 4 Clinical Toxicology Research Group, University of Newcastle, Newcastle, New South Wales, Australia; 5 Department of Biochemistry and Molecular Biology, Biomedicine Discovery Institute, Monash University, Melbourne, Victoria, Australia; 6 Department of Biochemistry, Faculty of Medicine, University of Peradeniya, Peradeniya, Sri Lanka; 7 Clinical Pharmacology, University of Sydney, Sydney, Australia; Liverpool School of Tropical Medicine, UNITED KINGDOM

## Abstract

**Background:**

Sri Lankan Russell’s viper (*Daboia russelii*) envenoming is reported to cause myotoxicity and neurotoxicity, which are different to the effects of envenoming by most other populations of Russell’s vipers. This study aimed to investigate evidence of myotoxicity in Russell’s viper envenoming, response to antivenom and the toxins responsible for myotoxicity.

**Methodology and Findings:**

Clinical features of myotoxicity were assessed in authenticated Russell’s viper bite patients admitted to a Sri Lankan teaching hospital. Toxins were isolated using high-performance liquid chromatography. *In-vitro* myotoxicity of the venom and toxins was investigated in chick biventer nerve-muscle preparations. Of 245 enrolled patients, 177 (72.2%) had local myalgia and 173 (70.6%) had local muscle tenderness. Generalized myalgia and muscle tenderness were present in 35 (14.2%) and 29 (11.8%) patients, respectively. Thirty-seven patients had high (>300 U/l) serum creatine kinase (CK) concentrations in samples 24h post-bite (median: 666 U/l; maximum: 1066 U/l). Peak venom and 24h CK concentrations were not associated (Spearman’s correlation; p = 0.48). The 24h CK concentrations differed in patients without myotoxicity (median 58 U/l), compared to those with local (137 U/l) and generalised signs/symptoms of myotoxicity (107 U/l; p = 0.049). Venom caused concentration-dependent inhibition of direct twitches in the chick biventer cervicis nerve-muscle preparation, without completely abolishing direct twitches after 3 h even at 80 μg/ml. Indian polyvalent antivenom did not prevent *in-vitro* myotoxicity at recommended concentrations. Two phospholipase A_2_ toxins with molecular weights of 13kDa, U1-viperitoxin-Dr1a (19.2% of venom) and U1-viperitoxin-Dr1b (22.7% of venom), concentration dependently inhibited direct twitches in the chick biventer cervicis nerve-muscle preparation. At 3 μM, U1-viperitoxin-Dr1a abolished twitches, while U1-viperitoxin-Dr1b caused 70% inhibition of twitch force after 3h. Removal of both toxins from whole venom resulted in no *in-vitro* myotoxicity.

**Conclusion:**

The study shows that myotoxicity in Sri Lankan Russell’s viper envenoming is mild and non-life threatening, and due to two PLA_2_ toxins with weak myotoxic properties.

## Author Summary

There are many gaps in our knowledge of muscle damage caused by snake venoms. Russell’s vipers are more medically important than any other snake in Asia. Sri Lankan Russell’s viper (*Daboia russelii*) bites have been reported to cause muscle damage in humans, which is not reported for other Russell’s vipers. The aim of the present study was to investigate the onset, severity and resolution of the muscle damage and to identify the toxins responsible for myotoxicity. For this, we studied muscle damage in 245 patients with confirmed Sri Lankan Russell’s viper bites. Patients reported local muscle pain in 72% of cases and generalised muscle pain in 15%. None had severe muscle damage and the symptoms resolved in 80% of patients within 4 days. Measurement of biomarkers of muscle damage in patient blood was consistent with only mild muscle injury, even in patients with symptoms. Two toxins were isolated from Sri Lankan Russell’s viper venom that had similar myotoxic activity to whole venom in chick muscle preparations. Both toxins were weak myotoxins, consistent with what was seen in patients.

## Introduction

Snake bite is a significant public health issue in the tropics [1]. Coagulopathy, neuromuscular paralysis, acute kidney injury and local effects are the most important clinical syndromes of snake envenoming [2]. Russell’s viper bites cause a large number of envenomings across Asia, and are more medically important than any other snake in the region [3,4]. Both species of Russell’s vipers, i.e. *D*. *russelii* (found in Sri Lanka, India, Pakistan, Nepal, Bangladesh) and *D*. *siamensis* (found in some parts of southeast and east Asia such as Cambodia, Myanmar, Thailand, Taiwan, South China and, some parts of Indonesia including East Java), commonly cause venom-induced consumption coagulopathy, acute kidney injury and mild local effects throughout their distribution [3,5]. However, there is geographical variation in the clinical effects of Russell’s viper envenoming with neuromuscular paralysis and rhabdomyolysis only reported from Sri Lanka and South India [3,6–9]. In a recent clinical and neurophysiological investigation of neuromuscular paralysis in Sri Lankan Russell’s viper envenoming, we showed that the paralysis is mild, non-life threatening with no long term effects [6]. Clinical evidence of myotoxicity, including local and generalized myalgia, muscle tenderness, and dark red or black coloured urine suggestive of myoglobinuria, has been reported in cases of Russell’s viper envenoming in Sri Lanka [7–10]. Elevation of plasma and urinary myoglobin concentrations were reported in 19 Russell’s viper bite patients from Sri Lanka further suggesting the existence of myotoxicity in Sri Lankan Russell’s viper patients [10]. A recent study of Sri Lankan Russell’s viper venom injected into mice reported an elevation in creatine kinase, also suggesting that the venom is myotoxic *in-vivo* [11].

Myotoxicity is an important effect of snake envenoming and can manifest locally and systemically [12]. Local muscle necrosis is a component of the local necrotic effects [12,13]. Systemic myotoxicity has been reported following envenoming by some vipers [14,15], sea snakes [16–18], Australasian elapids [19,20] and some Asian kraits [21,22]. Systemic myotoxicity ranges in severity. Loss of functioning muscle cells due to myotoxicity can aggravate co-existing weakness due to neuromuscular block caused by neurotoxins. More importantly rhabdomyolysis can cause secondary acute kidney injury [23,24] and itself can result in life-threatening hyperkalaemia due to extensive muscle cell damage [15].

Although many cytotoxic components in snake venoms may contribute to the development of myotoxicity, the most important snake venom myotoxins are phospholipase A_2_ (PLA_2_) toxins [12,25]. Three types of PLA_2_ myotoxins, commonly referred as Asp49, Ser49 and Lys49 PLA_2_, have been characterised in viperid venoms. Despite structural similarity, the latter two types lack catalytic activity, and are referred to as ‘PLA_2_ like’ peptides [26–28]. It is important to note that the enzymatic activity and the myotoxic activity of PLA_2_ myotoxins are independent [29]. Myotoxic PLA_2_s cause muscle damage primarily by destruction of the sarcolemma [12,30]. Several PLA_2_ toxins have been isolated from Sri Lankan Russell’s viper venom and biochemically characterised, [31,32] Some of these PLA_2_ toxins contain a unique Serine residue at the N-terminus (S-type), while others have an Asparagine residue at the N-terminus (N-type) [32]. We have recently shown that the pre-synaptic neurotoxicity of the Sri Lankan Russell’s viper venom is primarily due to one of the S-type PLA_2_ toxins, which we named U1-viperitoxin-Dr1a [33].

There are several gaps in our understanding of the myotoxicity associated with Sri Lankan Russell’s viper envenoming. It is unclear if Sri Lankan Russell’s viper envenoming causes severe myotoxicity and whether any resultant myotoxicity can be treated with Indian Polyvalent antivenom. Further, the *in vitro* myotoxicity of Sri Lankan Russell’s viper venom has not been pharmacologically studied. This requires isolation and pharmacological characterisation of the myotoxins from the venom. The present study aims to investigate the clinical severity of myotoxicity from Russell’s viper envenoming and isolate the toxins responsible for this activity.

## Methods

### Clinical study

#### Ethics, study setting and patients

We carried out a prospective cohort study of confirmed Russell’s viper bite patients aged over 16 years admitted to the Teaching Hospital Anuradhapura, Sri Lanka for a 14 month-period. The hospital is a tertiary care hospital which admits over 1000 suspected snake bite cases annually. Ethics clearance for this study was granted by the Human Research Ethics Committees of the University of Peradeniya (Approval No: 2012/EC/63), Sri Lanka, Rajarata University of Sri Lanka (Approval No: ERC 2013/019) and Monash University, Australia (Approval No: CF14/970–2014000404). Written informed consent was obtained prior to the recruitment of all patients. For patients 16 and 17 years of age, consent was also obtained from the patient’s parent or guardian. The recruitment of patients to this cohort study has been previously described. [6]

#### Case authentication

Cases were authenticated by either positive identification of the snake specimen involved as a Russell’s viper by AS, who is a herpetologist, or by detection of the specific Russell’s viper venom antigens in blood samples of the patients using enzyme-immunoassay (EIA).

#### Clinical data collection and blood collection

Clinical examination of all patients was undertaken on admission to hospital, 1 h, 4 h, 8 h, 12 h and 24 h post-bite, and then every 24 h until discharge. In particular, muscle pain and tenderness, in the bitten limb (local myotoxicity) and the other limbs (generalized myotoxicity) were assessed as part of the clinical examinations. Blood samples were collected from patients on admission and then 1 h, 4 h, 8 h, 12 h and 24 h post-bite, and daily thereafter. All blood samples were immediately centrifuged, serum aliquoted and frozen initially at -20°C before transfer to -80°C storage until they were analysed.

#### Antivenom

Indian polyvalent antivenoms from VINS Bioproducts, India (batch numbers: 01AS11118, 1119, 1121, 1123, 3100, 4001, 4025, 4026, 4031) and BHARAT Serums and Vaccines Ltd, India (batch number: A5311018) were used throughout the study period. In the present cohort, the majority of patients received antivenom and a small proportion of these received their first dose of antivenom at a primary care hospital, before being transferred to the study hospital. The decision to administer antivenom was made solely by the treating physician, based on clinical and laboratory evidence of systemic envenoming. Patients were administered between 10 to 20 vials of antivenom which is the standard dose. Antivenom was administered in normal saline and infused over 1h. If an acute adverse reaction to antivenom occurred, the infusion was stopped for 5 to 10 min and the patient treated with adrenaline, antihistamines and corticosteroids, as per the treating clinicians.

#### Russell’s viper venom enzyme immunoassays

Measurement of venom in the samples was carried out using a Russell’s viper venom specific Enzyme-Immunoassay (EIA) which has been previously described [6,34,35]. In brief, rabbit IgG was raised against Russell’s viper venom from Sri Lanka. Antibodies were bound to microplates as well as conjugated to biotin as the detecting antibodies with streptavidin-horseradish peroxidase. The lower limit of detection for the assay was 2.5 ng/ml. In cases where no venom was detected in pre-antivenom samples or pre-antivenom samples were not available, the post-antivenom samples were subjected to heat dissociation treatment and then tested for venom using the same EIA [35].

#### Serum creatine kinase (CK) assay

Creatine kinase (CK) was measured using the Thermo Scientific Ltd CK-NAC reagent kit on stored frozen serum samples. Patient serum samples were thawed and 15 μL added to 300 μL of reagent. After 120 s, the samples were read in a plate reader (340 nm, 37°C) for 3 min. An abnormal CK was defined as a concentration greater than 300 U/l. A screening assay was done on all 24 h post-bite samples, or as close to this time point as possible. If the screening assay was abnormal (> 300 U/L), then serial CK assays were carried out for all samples available for that patient.

#### Data analysis

Continuous variables were reported as medians, range and interquartile range (IQR). Associations between venom concentration and CK measurements were assessed by Spearman’s correlation test. The CK concentrations between groups of patients was compared with the Kruskal-Wallis test. Analysis of clinical data was done using PRISM, version 6.05 (GraphPad Software, Inc.).

### Pharmacological study

#### Venom and antivenom

Freeze-dried Russell’s viper (*D*. *russelii*) venom from Sri Lanka (pooled from both sexes, including juveniles) donated by Professor Ariaranee Gnanadasan (Faculty of Medicine, University of Colombo, Sri Lanka) was used for this study. Venom was dissolved in MilliQ water and stored at -20°C until required. Protein quantification of the venom, fractions and toxins was carried out using a BCA Protein Assay Kit (Thermo Fisher Scientific, Rockford, IL, USA), as per the manufacturer’s instructions. The relative abundance of the two isolated toxins was determined by the area under the chromatogram of the whole venom. Isolation of the two toxins was performed as described, from whole venom, using the two chromatographic techniques. Here, the two toxins separately and the venom without the two toxins were collected by multiple HPLC runs and pooled separately. Then, the three samples (i.e. venom without both toxins, and the two toxins) were dissolved separately. Based on the protein assay, the protein/toxin amounts in the samples were determined. Then the different combinations of the venom with and without individual toxins were made by mixing the venom without the two toxins with appropriate toxin according to the proportion present in the venom.

Indian polyvalent antivenom (VINS Bioproducts, Thimmapur, India; batch No: 01AS14035) was used for the study. The antivenom was reconstituted in 2 mL of sterile injection water. According to the manufacturer, 1/10 of the antivenom in a single vial neutralises 0.6 mg of Russell’s viper venom. For *in-vitro* studies examining the neutralisation of myotoxins, the required amount of antivenom was calculated based on the relative abundance of the toxin in the venom.

#### In-vivo myotoxicity studies

Male Sprague–Dawley rats (300-360g) were anaesthetised with sodium pentobarbital (60–100 mg/kg, i.p.) with additional anaesthetic being administered during the experiment as required. An incision was made in the cervical region, the carotid artery was cannulated for blood sample collection and the trachea intubated for artificial respiration, if required. The rats were kept on small animal surgery tables with their body temperature maintained at 37°C. Venom (250 μg/kg in 50 μl 0.9% saline, n = 4) was injected into the gastrocnemius muscle of the left hind-limb. For control rats (n = 4), 50 μl 0.9% saline was injected in the same location. Blood samples (500 μl) were collected into MiniCollect LH/Gel separation tubes (Greiner bio-one), through the carotid cannula immediately before the venom/saline injection and at 1.5, 3, 4.5 and 6 h post-injection. At 6 h, rats were humanely killed with an overdose of sodium pentobarbitone. For the baseline measurements of CK, blood samples of an additional seven ‘control’ (i.e. not envenomed) rats were used. Measurement of CK was done using the same CK assay method used for human samples.

#### Liquid chromatography

Fractionation was performed using a Shimadzu (Kyoto, Japan) system (LC-10ATvp pump and SPD-10AVP detector).

Size-exclusion chromatography: Crude venom (500 μg) was reconstituted in buffer A (ammonium acetate; 0.1 M, pH 6.8). Venom was then separated using a Superdex G-75 column (13 μm; 10 mm × 300 mm; GE Healthcare, Uppsala, Sweden) equilibrated with buffer A. Elution was performed at a flow rate of 0.5 ml/min and monitored at 280 nm. All fractions were freeze-dried immediately and later screened using the chick biventer cervicis nerve-muscle preparation (see 2.2.6 below) to identify those with myotoxicity.

Reverse-Phase HPLC (RP-HPLC): The fractions obtained from size exclusion chromatography, which displayed *in vitro* myotoxicity, were subjected to further analysis using reverse-phase HPLC. Size exclusion chromatography fractions were reconstituted in solvent A (0.1% trifluoroacetic acid [TFA]) and 2 mg of total protein was injected into a Phenomenex Jupiter semi-preparative C_18_ column (250 mm ×10 mm; 5 μm; 300 A˚), equilibrated with solvent A. Fractions were eluted using the following gradient of solvent B (90% Acetronitrile in 0.1% TFA): 0–30% over 10 min, 30–70% for 10–50 min, and 70–0% for 50–55 min at a flow rate of 2.0 ml/min.

#### Matrix-Assisted Laser Desorption/Ionization (MALDI-TOF) Mass Spectrometry

MALDI-TOF Mass Spectrometry analysis was performed in order to determine the intact mass of the toxins, with an Applied Biosystems (Forster City, CA, USA) 4700 TOF TOF Proteomics Analyser, as described previously (Chaisakul et.al.). [36]

#### Amino acid sequence determination by Liquid Chromatogarphy-Mass Spectrometry (LC-MS)

Freeze-dried U1-viperitoxin-Dr1b dissolved in 100 mM ammonium bicarbonate buffer, 2 mM DL-Dithiothreitol, incubated for 20 min at 65°C and alkylated with 5 mM chloroacetamide. The protein mixture was digested with trypsin at 1:100 (w/w) ratio for 18 h at 37°C. The resulting peptides were desalted with Pierce C18 Spin columns (ThermoFisher Scientific), dried, resuspended in 2% Acetronitrile, 0.1% TFA and analysed by LC-MS/MS analysis as previously described [37]. The raw data acquired by the mass spectrometer was converted into a centroided peaklist file using ProteoWizard (version 3.0.9967) [38]. The MS/MS results were analysed with PMI-Byonic software (Protein Metrics, ver 2.8.14) against Uniprot serpents (taxID:8570) protein sequences (update 07/2016) using the following search parameters: trypsin digestion with up to 2 missed cleavages, fixed cysteine alkylation, variable methionine oxidation and asparagine and glutamine deamidation, precursor mass tolerance 10 ppm and fragment mass tolerance 20ppm.

#### PLA_2_ activity

PLA_2_ activity of the venom and U1-viperitoxin-Dr1b was determined using a secretory PLA_2_ assay kit (Cayman Chemical, Ann Arbor, MI, USA) as described previously [33] and according to the manufacturer’s instructions.

#### Chick biventer cervicis nerve-muscle preparation

Male chickens (aged 4–10 days) were humanely killed by exsanguination following CO_2_ inhalation. Biventer cervicis nerve-muscle preparations were dissected and then mounted on wire tissue holders under 1 g resting tension in 5 ml organ baths. Tissues were maintained at 34°C, bubbled with 95% O_2_ and 5% CO_2_, in physiological salt solution of the following composition (mM); 118.4 NaCl, 4.7 KCl, 1.2 MgSO_4_, 1.2 KH_2_PO_4_, 2.5 CaCl_2_, 25 NaHCO_3_ and 11.1 glucose. Direct twitches were evoked by stimulating the muscle belly (rate: 0.1 Hz; pulse duration: 2 ms) at supramaximal voltage (20–30 V) with the two electrode loops directly encircling the muscle belly, using a Grass S88 stimulator (Grass Instruments, Quincy, MA). Contractile responses of the tissues to KCl (40 mM for 30 s) was obtained in the absence of muscle stimulation. Following that, direct stimulation of the muscle was resumed. Selective stimulation of the muscle was ensured by abolishment of any indirect twitches by the continual presence of adding d-tubocurarine (10 μM) to the organ bath. The preparations were then stimulated for 30min, before the addition of venom or toxins. For antivenom experiments, the tissues were equilibrated with antivenom for 20 min before the venom or toxin was added. The preparation was exposed to the treatment/control until abolishment of direct twitches or for a period of 3 h. At the conclusion of the experiment, KCl was re-added as above.

#### Data analysis and statistics

The quantity of the toxins in the venom was determined by measuring the area under the curve of the size-exclusion and RP HPLC venom elution profiles. Direct twitch responses and responses to KCl were measured via a Grass FTO3 force displacement transducer and recorded on a PowerLab system (ADInstruments Pty Ltd., Australia). Responses were expressed as percentages of their pre-venom/toxin values. T_90_ values (i.e. time taken for 90% inhibition of the maximum twitch response to occur) were determined for the two toxins. To compare the responses to exogenous agonists following the administration of venom a one-way ANOVA was used. All ANOVAs were followed by Bonferroni’s multiple comparison post-tests. Data are presented in the form of mean ± standard error of the mean (S.E.M.) of n experiments. All statistical analyses and presentation of data were generated using GraphPad Prism 6.05 software. For all statistical tests p < 0.05 was considered statistically significant.

#### Animal ethics

All animal experiments used in this study were approved by the Monash University Animal Ethics Committee (Approval No: MARP/2014/097).

## Results

### Myotoxicity in authenticated cases

#### Clinical features of myotoxicity

**Fig 1:**
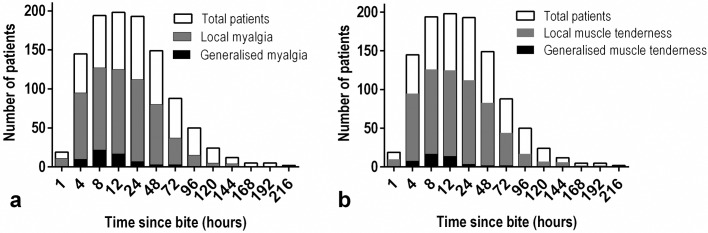
Clinical features of myotoxicity in 245 confirmed Sri Lankan Russell’s viper envenomings (a), the presence of localised and generalised myalgia over time; (b), the presence of localised and generalised muscle tenderness over time. Note: data of all 245 patients were not available at any given time because some patients were admitted later than the time point or discharged before the time point, or the clinical assessment of the patient was not possible at that time point because the patient was sedated, unconscious or transported away for investigations.

Of the 245 authenticated Russell’s viper bite patients recruited to the study, 177 (72.2%) had myalgia and 173 (70.6%) tenderness in the bitten limb (local myotoxicity). Thirty-five patients (14.2%) and 29 patients (11.8%) had generalized myalgia and generalized muscle tenderness, respectively. Changes in local and generalized myalgia and muscle tenderness over time among the study participants is shown in Fig 1A and 1B. Myalgia and muscle tenderness resolved in over 80% of the patients by day 4. Six patients (2.4%) had dark coloured urine observed between 12 and 48 h after the bite. The clinico-epidemiological data related this cohort of patients is described in the Table 1.

**Table 1:** Clinical and epidemiological data of the 245 patients recruited for the present study.

Age (Median, range in years)	41 (16–70)
Sex (males)	171 (70%)
Length of the snake specimen (median, range)	32.3 (20.4–110.5) cm
Time since bite to reach the study hospital (Median, IQR)	2.5 (1.75–4.0) h
Peak venom concentration (median, range)	25 (2.5–2316) ng/ml
Number of patients with coagulopathy	166 (68%)
Number of patients with neurotoxicity	130 (53%)
Number of patients received antivenom	190 (78%)
Time from snakebite to antivenom (Median, IQR)	3.75 (2.75–5.0) h
Number of antivenom vials received (median, range)	20 (10–90)
Length of hospital stay	3 (2–31) days

#### Serum creatine kinase concentrations

**Fig 2:**
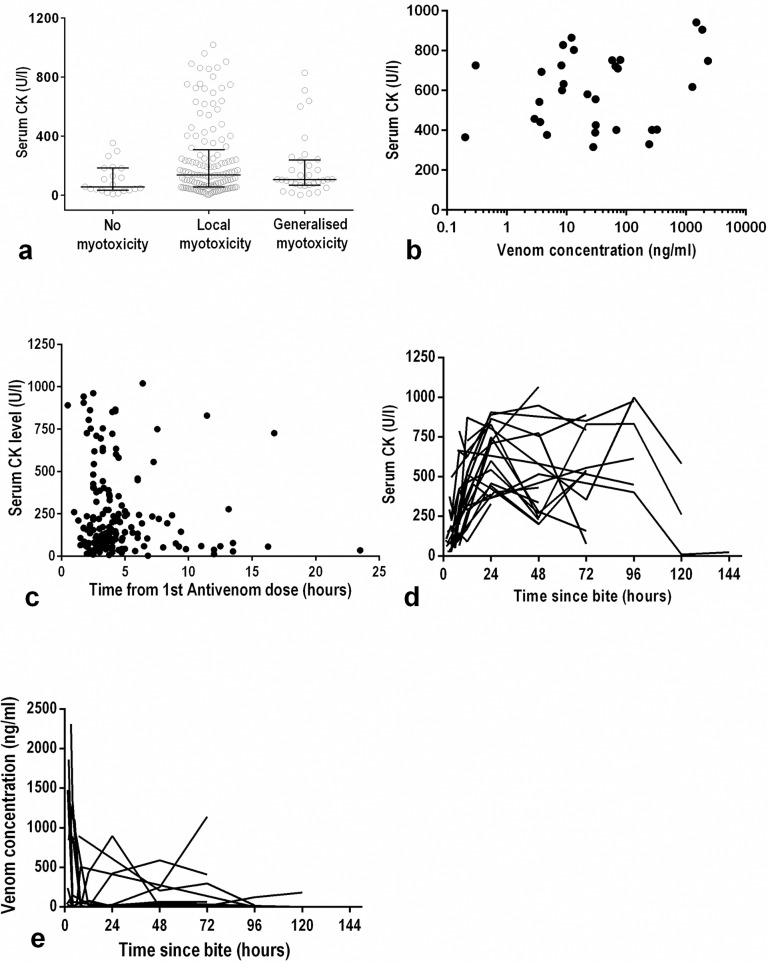
Serum creatine kinase (CK) concentrations in 219 study participants: (a) Scatter plot comparing the CK concentrations (at 24 h post bite) of patients with no features, patients with features of local myotoxicity (myalgia or tenderness) and patients with features of systemic myotoxicity (*p = 0*.*049; Kruskal-Wallis test*); (b), peak serum concentrations of CK versus venom in 37 patients who had CK >300U/l at 24 h post-bite (*Spearman’s correlation; p = 0*.*48*); (c), CK concentration at 24 h versus time from the snakebite to the first antivenom dose in 178 patients who received antivenom (note: 12 patients who received antivenom had no CK due to the unavailability of the sample at 24 h); (d), plots of the CK concentrations versus time since bite for the 37 patients; (e), plots of the venom concentrations versus time for the 37 patients.

Serum CK concentrations in 24 h post-bite samples of 219 patients ranged from 2 to 1019 U/l (median: 110 U/l). Samples of the remaining 26 patients were unavailable. Of the 219 patients, 37 patients (16.8%) had CK concentrations above 300 U/l. The median 24 h post-bite CK concentrations of patients who had no clinical features of myotoxicity was 58 U/l (IQR: 34–183 U/l), compared to 137 U/l (IQR: 56–285 U/l) in those with features of local myotoxicity, and 107 U/l (IQR: 71–226 U/l) in patients who had features of systemic myotoxicity (p = 0.049; Kruskal Wallis test; Fig 2A). There was no statistically significant association between 24 h serum CK concentration and peak venom concentrations (Spearman’s correlation; p = 0.48; Fig 2B). Similarly, there was no relationship between the time delay from the snakebite to first dose of antivenom and 24 h serum CK (Fig 2C). Of the 190 patients who were given antivenom, CK concentrations at 24 h were available in 178 patients. Of these, 135 patients were given antivenom within 6 h and 31 of these developed a CK > 300 U/l at 24 h, compared to 31 patients who received antivenom after 6 h with 6 developing a CK > 300 U/l at 24 h.

Only three of the six patients with dark coloured urine had CK concentrations above 300 U/l (404, 633 and 856 U/l,). However, red blood cells were reported in three of these six patients (S1 Table). The dark coloured urine in Russell’s viper envenoming could be due to myoglobinuria, haemoglobinuria, haematuria or a combination of all these.

The serial CK concentrations of the 37 patients with high CK had a median peak value of 666 U/l (maximum: 1066). In most patients, CK reached a peak in 24 h after the bite (Fig 2D). By the time CK started to rise, the venom concentrations in these patients had already decreased, and in most cases was lower than the level of detection (Fig 2E).

### *In-vivo* myotoxic effects in anaesthetised rats

The plasma CK concentrations of rats injected with Sri Lankan Russell’s viper venom 250 μg/kg (i.m.) were not different tocontrol rats and were within the normal range until 6 h of the venom injection (S1 Fig).

### Isolation and biochemical characterisation of U1-viperitoxin-Dr1a and U1-viperitoxin-Dr1b

#### Liquid Chromatography

**Fig 3:**
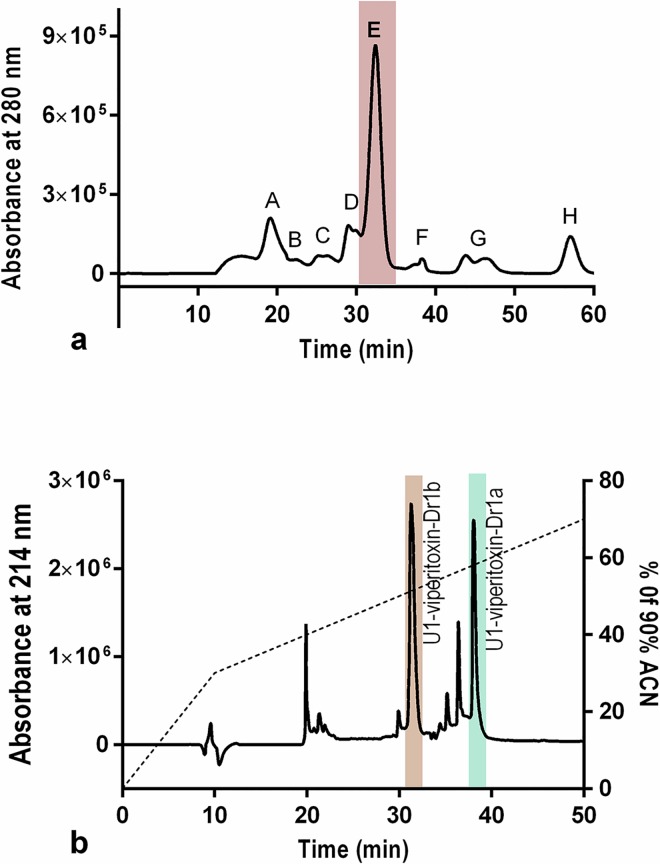
Chromatograms of Sri Lankan Russell’s viper venom and fractions (a), Size-exclusion chromatogram of the whole venom on Superdex G75 column; (b), Reverse Phase High Performance Liquid Chromatogram (RP-HPLC) of the fraction ‘E’ on Jupiter C_18_ semi-preparative column. Note: the peaks eluting at 31 and 38 min in RP-HPLC chromatograms are U1-viperitoxin-Dr1b and U1-viperitoxin-Dr1a, respectively.

**Fig 4:**
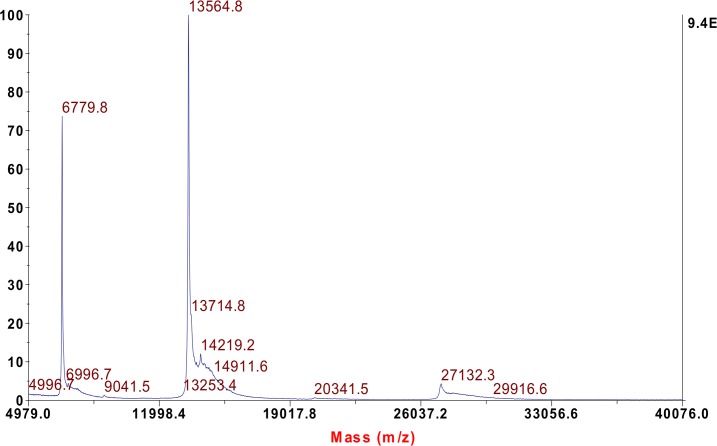
Intact protein analysis chromatogram of MALDI-TOF: the intact mass of U1-viperitoxin-Dr1b is 13.564 kDa.

Eight major fractions were identified with size-exclusion chromatography of the crude venom of Russell’s viper (Fig 3A). Peak E, eluting around 33 min showed marked myotoxicity in the chick biventer cervicis nerve-muscle preparation. Peak E was further analysed and fractionated using RP-HPLC (Fig 4B). *In vitro* myotoxicity was largely confined to two peaks with retention times of 31 min and 38 min, respectively (Fig 3B). The former venom component eluting at 31 min was subsequently isolated as U1-viperitoxin-Dr1b, and constituted 22.7% (SD: 1.2; n = 10) of the crude venom, based on the area under the curve of the size exclusion chromatography and RP-HPLC profiles of the venom. The component eluting at 38 min was named U1-viperitoxin-Dr1a, a PLA_2_ toxin which has been previously characterised biochemically and found to be the major neurotoxin (with a relative abundance of 19.2%) in Sri Lankan Russell’s viper venom [33].

### Intact protein analysis of U1-viperitoxin-Dr1b by MALDI-TOF

Analysis of U1-viperitoxin-Dr1b by MALDI-TOF mass spectrometry indicated a single species with a mass/charge ratio of 13564 Da (Fig 4). A doubly charged ion with a mass/charge ratio of 6779.8 Da was also observed.

#### LC-MS/MS identification of U1-viperitoxin-Dr1b by LC-MS

LC-MS/MS analysis of U1-viperitoxin-Dr1b identified it as Basic phospholipase. The obtained sequence coverage was 100% showing that the toxin is exactly identical to Basic phospholipase A_2_ VRV-PL-VIIIa (SwissProt: **P59071**, Entry name: PA2B8_DABRR) (see S2 Fig and S2 Table).

#### PLA_2_ activity of U1-viperitoxin-Dr1b

PLA_2_ activity of U1-viperitoxin-Dr1b was 1139 (+/- 19) μmol/min/mg.

#### Pharmacological investigation of the myotoxicity

**Fig 5:**
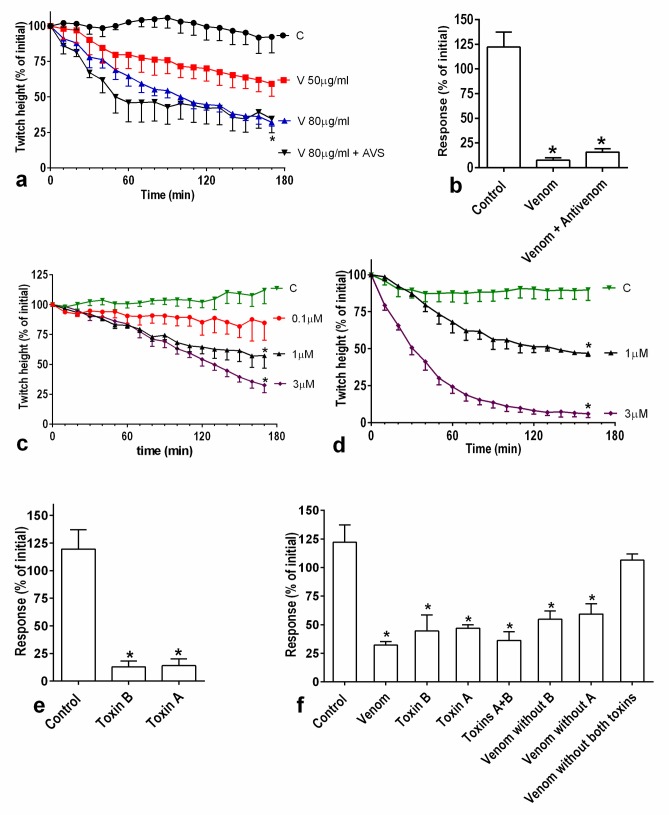
*In-vitro* myotoxicity of whole venom (Venom) of Sri Lankan Russell’s viper, U1-viperitoxin-Dr1a (Toxin A) and U1-viperitoxin-Dr1b (Toxin B) compared to controls: **(a)**, Concentration-dependent inhibition of direct twitches in chick biventer nerve-muscle preparation by whole venom. (* the 80μg/ml venom group is significantly different from 50 μg/ml group as well as the control group at 170 min; *p<0*.*05*: *One-way ANOVA followed by Bonferroni’s post-hoc test*, *n = 4*.). **(b),** Effect of 80μg/ml venom alone and in the presence of antivenom on the response of the muscle to 40mM KCl. (* significantly different compared to the response to KCl obtained prior to the addition of venom; *p<0*.*05*: *paired t-test*) **(c)**, Concentration-dependent inhibition of the direct twitches in chick biventer nerve-muscle preparation by U1-viperitoxin-Dr1b. *(twitch height significantly lower than the control group: *p<0*.*05*, *one-way ANOVA followed by Bonferroni’s post-hoc test; n = 3–5*); **(d)**, Concentration-dependent inhibition of the direct twitches in chick biventer nerve-muscle preparation by U1-viperitoxin-Dr1a. (* twitch height significantly lower than the control group: *p<0*.*05*, *one-way ANOVA followed by Bonferroni’s post-hoc test; n = 3–5*); **(e)**, Effect of 3 μM U1-viperitoxin-Dr1a (B) and U1-viperitoxin-Dr1b (M) towards the response of the muscle for 40 mMKCl. (* the KCl response of the venom with and without antivenom, at 170min were significantly reduced compared to the initial responses; *p<0*.*05*: *paired t-test*); **(f)**, Effect of 50μg/ml whole venom versus U1-viperitoxin-Dr1a, U1-viperitoxin-Dr1b, venom without U1-viperitoxin-Dr1a, venom without U1-viperitoxin-Dr1b, both toxins together, venom without both toxins of a same amount of venom in 5ml organ bath towards the response of the muscle for 40 mMKCl. * (* the KCl response is significantly lower compared to the control group at 170 min; *p<0*.*05*: *one-way ANOVA followed by Bonferroni’s post-hoc test*; n = 3–5).

Sri Lankan Russell’s viper venom caused concentration-dependent inhibition of direct twitches in the chick biventer nerve muscle preparation (Fig 5A) and inhibited the response to 40 mM KCl (Fig 5B). However, high venom concentration such as 80μg/ml did not completely abolish either the direct twitches or the response to KCl, even after 3 h. U1-viperitoxin-Dr1b (Fig 5C) and U1-viperitoxin-Dr1a (Fig 5D) concentration-dependently inhibited the direct twitches and also inhibited the response to KCl (Fig 5E). When the response to KCl was compared between the crude venom 50μg/ml, each of the two toxins of the same amount of venom individually, a combination of the two toxins, and the whole venom without individual or both toxins, removal of the two toxins led to a loss of all myotoxicity from the venom (Fig 5F). Antivenom, at the recommended concentrations, did not neutralise the myotoxic effect of the venom (Fig 5B)

## Discussion

The present study demonstrates that myotoxicity seen in humans following Sri Lankan Russell’s viper envenoming is mild, based on the relatively low frequency of generalized myotoxic features and only a very mild elevation in CK. There were small but statistically significant association between clinical features of myotoxicity and the 24 h CK measurements. The rats injected intramuscularly with Sri Lankan Russell’s viper venom did not show an elevation of CK within the first 6 h. Although Sri Lankan Russell’s viper venom displays concentration-dependent myotoxic effects *in-vitro*, very high venom concentrations (i.e. >50 μg/ml) are required to completely inhibit direct twitches in the chick biventer nerve-muscle preparation and the response to KCl. Antivenom at recommended concentrations was unable to neutralise the myotoxic properties of the venom *in-vitro*. The myotoxicity of the whole venom appears to be due to two abundant PLA_2_ toxins in the venom, U1-viper1toxin-Dr1a and U1-viperitoxin-Dr1b (named herein), which both have *in vitro* myotoxic activity, but only at very high concentrations (3 μM).

Features of systemic myotoxicity such as generalized muscle tenderness and myalgia, and dark coloured urine were observed in less than 15% of the patients. In addition, three of the six patients with dark coloured urine did not have 24 h CK concentrations outside the normal limit, so the dark urine was more likely due to macroscopic haematuria (due to haemorrhage) which occurs with Russell’s viper envenoming. Peak CK concentrations were abnormally high in less than 17% of patients, and even in these cases there were only modest elevations with the median and maximum being 666 and 1066 U/l, respectively. In comparison, the reported median peak CK values following envenoming by mulga snakes and tiger snakes in Australia were 3100 U/l [20] and 4749 U/l [19], with severe myotoxicity being associated with CK values over 100,000 U/L. This means that myotoxicity in Russell’s viper envenoming is uncommon and mild when it occurs compared to other myotoxic snakes. The median 24 h CK concentrations were higher in symptomatic patients demonstrating that the clinical features were consistent with biochemical evidence of mild muscle injury.

Anaesthetised rats injected with venom showed no significant increase in serum CK concentrations compared to saline injected rats, even 6 h after the venom injection. Assuming that the blood volume of a rat with a body weight of 330g is approximately 25 ml, the maximum serum venom concentration that this venom dose (i.e. 250 μg/kg), with 100% systemic absorbance, could give rise to is 3300 ng/ml. This venom concentration is greater than the maximum venom concentration observed in the Russell’s viper envenomed patients in this study, hence the venom dose used for the envenoming is clinically relevant. The absence of a significant CK rise in the envenomed rats during the 6 h observation period may be due to a combined effect of the weak myotoxicity of the venom and the period of observation being too short to observe any CK elevation. These results are not in agreement with the previously reported high CK concentrations (mean: 16,000 U/l) in mice, 3 h after the intramuscular injection of 5 μg (250 μg/kg dose in 20 g mouse) of Sri Lankan Russell’s viper venom [11], despite similar venom doses in the two studies. The reason for the discrepancy is unclear but such a large early increase in CK concentrations is unusual and the mouse model has not previously been validated.

The clinical findings were consistent with the *in vitro* studies in which two low potency myotoxins were found to be relatively abundant in the venom. Very high venom concentrations up to 80 μg/ml (80 times higher than the maximum peak venom concentration observed in envenomed patients) were required to decrease the direct twitch force by two thirds within 3 h. The low potency of both myotoxins was evident from the fact that high concentrations (3 μM) were required for significant *in-vitro* myotoxic activity. Venom with these two toxins removed did not cause myotoxicity confirming that these toxins are the only major myotoxins in Sri Lankan Russell’s viper venom.

Three previous studies between 1984 and 2000 report much higher rates of myotoxicity, based mainly on myalgia and muscle tenderness. In 1984, Jeyarajah [9] reported myotoxic symptoms in 77% of the cases but no biochemical confirmation of myotoxicity. This study included only severe envenoming with 19/22 with acute kidney injury and 6 deaths. In 1988, Phillips et. al. [10] reported myotoxic symptoms in 32% of the cases, but again this only included more severe envenoming (4/23 died). The mean venom concentration of 375 ng /ml (range: 16.5–702 ng/ml) [10] was much higher compared to the median peak venom concentration of 25 ng/ml (range:2.5–2316 ng/ml) in our study [6]. Phillips et al. reported serum and urine myoglobin, which are difficult to interpret because no studies have correlated these measurements with outcomes. They detected myoglobin in the plasma of all 19 patients tested (range: 100->8000; median: 2745, normal value:<50 ng/ml) and in the urine in 14 of 18 patients (110 to >16 000; median: 4000; normal: <21 ng/ml)[10]. Although these appear to be high, much higher values are reported in Australian mulga snake envenoming, where urinary myogolobin values in three patients were 4129, 28200 and 127000 ng/ml[20]. In a larger study of 336 patients in 2000 by Kularatne et al [7] there were 47 patients (14%) with myotoxicity based on generalised muscle tenderness. This is consistent with our study in terms of clinical effects but they did not provide any biochemical confirmation. Our 245 patients are typical of current patients. They had high circulating peak venom concentrations with a median of 25ng/ml, with 24 patients having venom concentrations >1000 ng/ml [6]. Further, nearly all had local envenoming, 68% had coagulopathy (half with bleeding manifestations), and 53% had neuromuscular paralysis [6]. Therefore, the mild myotoxic effects in Sri Lankan Russell’s viper envenoming are most likely due to the weak myotoxicity of the venom.

In patients with Australian Mulga snake (*Pseudechis australis*) envenoming, a delay in antivenom treatment has led to an increase in the severity of the myotoxicity [20]. Since there was no control group of patients who did not receive antivenom in this study, it could be argued that the weak myotoxicity observed in Sri Lankan Russell’s viper envenoming is due to an effect of antivenom. However, there was no association between the delay in antivenom treatment and the number of patients having CK >300 U/l, indicating that the weak myotoxicity observed in the patients is not due to an effect of the antivenom.

Indian Polyvalent antivenom was used at concentrations equivalent to the tested venom as recommended by the manufacturer. At these concentrations antivenom failed to prevent the myotoxic effects of Sri Lankan Russell’s viper venom *in-vitro*. Although this may be due to the low efficacy of the antivenom against the myotoxins [39], testing higher antivenom concentrations was not possible due to the practical limitations of increasing antivenom concentration in the tissue organ bath environment without affecting the osmolarity of the physiological salt solution. It was therefore not possible to determine the efficacy of the Indian polyvalent antivenom to neutralise the myotoxic effects of Sri Lankan Russell’s viper venom, because such a large amount of venom was required to cause myotoxicity requiring very high concentrations of antivenom.

**Fig 6:**
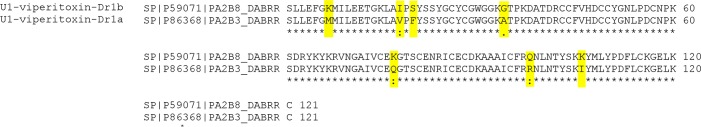
Alignment of U1-viperitoxin-Dr1b amino acid sequence with U1-viperitoxin-Dr1a. Sequences were obtained from UniProt database and are presented with unique identification numbers and entry names. In residues marked as ‘*’ are single fully conserved residues. At the positions highlighted in yellow, ‘:’ and ‘.’ denote positions with conservation between groups of strongly similar properties (scoring > 0.5 in the Gonnet PAM 250 matrix) and, conservation between groups of weakly similar properties (scoring = < 0.5 in the Gonnet PAM 250 matrix) respectively.

Of the several ‘s’ type PLA_2_ toxins isolated from Sri Lankan Russell’s viper venom, VRV-PL-VIIIa [40] has 100% match with the aligned trypsin digested peptide fragments of U1-viperitoxin-Dr1b. The N-terminal sequence of the first 50 amino acids of the toxin ‘P1’ [31] and the N-terminal sequence of first 21 amino acids of the toxin PLA_2_ 4 [32] are a 100% match for the sequence of U1-viperitoxin-Dr1b. Following the suggested rational nomenclature for toxins [41], and given that the 94% sequence homology of the toxin with U1-viperitoxin-Dr1a[33], we have named the above toxin as U1-viperitoxin-Dr1b (Fig 6).

The recent study on the venom proteome of the Sri Lankan Russell’s viper [11] reported that five PLA_2_ toxins make up a relative abundance of 35% of the whole venom, with VRV-PL-VIIIa (U1-viperitoxin-Dr1b) making 13.9% (as opposed to 22.2% in our study) of the whole venom. However, the same study did not match a PLA_2_ with VRV-PL-V (U1-viperitoxin-Dr1a), which makes 19.2% of the venom in our analysis as previously published [33].

In conclusion, myotoxicity in Sri Lankan Russell’s viper envenoming is mild and non-life threatening, as evident from the low frequency of generalized myotoxicity and low concentrations of serum creatine kinase in envenomed patients. The whole venom of Sri Lankan Russell’s viper has weak myotoxic properties *in-vitro*. Two PLA_2_ toxins, U1-viperitoxin-Dr1a and U1-viperitoxin-Dr1b that make 42% of the whole venom, are the major myotoxins in the venom, but display weak myotoxicity. There is a small possibility that myotoxicity may occur in patients with severe Sri Lankan Russell’s viper envenoming in which antivenom is delayed.

## Supporting Information

S1 Checklist checklist of the guidelines for Strengthening the Reporting of Observational Studies in Epidemiology (STROBE). (DOC)

S1 Table Evidence of muscle damage, renal injury and haematuria in six patients who had dark coloured urine. (DOCX)

S2 Table LC-MS/MS based identification of the amino acid sequence of U1-viperitoxin-Dr1b: List of all the identified proteins and the matched peptides of each proteins with the identification-related information. (XLSX)

S1 Fig Plasma CK concentrations in anaesthetised rats (n = 4) intramuscularly injected with 250 μg/kg Sri Lankan Russell’s viper venom compared to the saline injected rats (n = 4). Note: there is no difference in the CK concentrations of the two groups. The shaded area indicates the baseline CK concentration observed in 15 rats.
(TIF)

S2 Fig U1-viperitoxin-Dr1b protein coverage map based on MS/MS identification. All U1 Dr1b tryptic peptides that were identified by MS/MS ion search are marked as green lines and aligned according to their start and end position along U1 Drb1 sequence (shown on top). Modified residues are shown in red.
(JPG)
