# Supplementary material for: Clinical and Pharmacological Investigation of Myotoxicity in Sri Lankan Russell’s Viper (Daboia russelii) Envenoming
Source: PLoS Negl Trop Dis. 2016 Dec 2;10(12):e0005172. doi: 10.1371/journal.pntd.0005172 (PMC5135039; doi:10.1371/journal.pntd.0005172)
Supplement: S1 Table — (DOCX) [file pntd.0005172.s002.docx]

|  | Peak Serum creatinine kinase value (IU/l) | Highest Serum creatinine value (μmol/l) | Oliguria | Haemodyalysis or peritoneal dialysis performed? | Evidence of haematuria in urine full report |
| --- | --- | --- | --- | --- | --- |
| Patient 1 | 28 | 126 | No | No | Red cells 4-6 per hpf |
| Patient 2 | 58 | 139 | Yes | Yes | nil |
| Patient 3 | 100 | 179 | No | No | nil |
| Patient 4 | 404 | 844.9 | Yes | Yes | Red cells 5-16 per hpf |
| Patient 5 | 633 | N/A | No | No | nil |
| Patient 6 | 856 | 686.5 | Yes | Yes | Red cells 4 -10 per hpf |

Supplementary table
