# Supplementary figures and images for: Clinical and Pharmacological Investigation of Myotoxicity in Sri Lankan Russell’s Viper (Daboia russelii) Envenoming
Source: PLoS Negl Trop Dis. 2016 Dec 2;10(12):e0005172. doi: 10.1371/journal.pntd.0005172 (PMC5135039; doi:10.1371/journal.pntd.0005172)

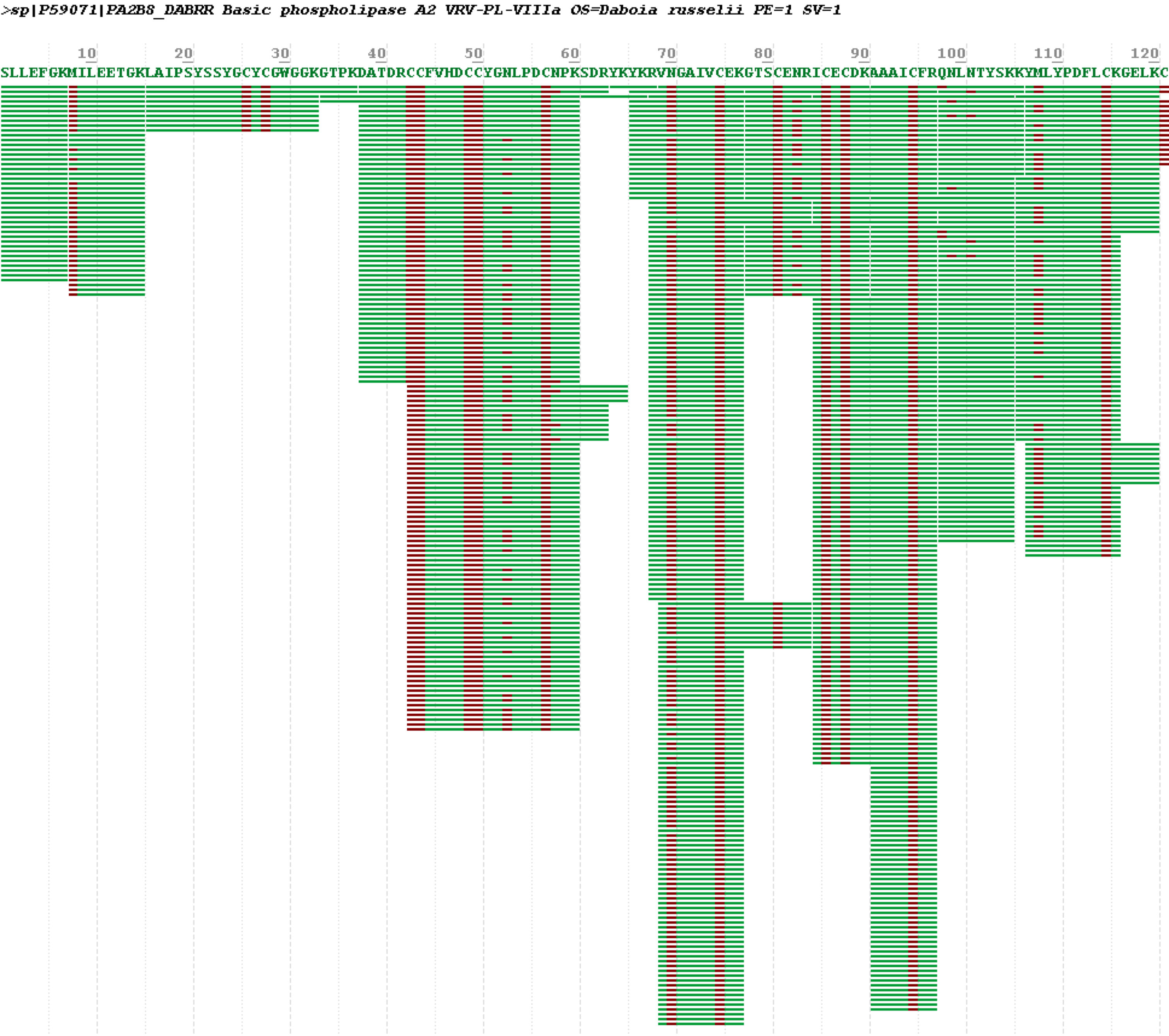

Supplement: S2 Fig — All U1 Dr1b tryptic peptides that were identified by MS/MS ion search are marked as green lines and aligned according to their start and end position along U1 Drb1 sequence (shown on top). Modified residues are shown in red. (JPG) [file pntd.0005172.s005.jpg]
